# Transcriptional profiling of Epstein–Barr virus (EBV) genes and host cellular genes in nasal NK/T-cell lymphoma and chronic active EBV infection

**DOI:** 10.1038/sj.bjc.6602968

**Published:** 2006-01-31

**Authors:** Y Zhang, J H Ohyashiki, T Takaku, N Shimizu, K Ohyashiki

**Affiliations:** 1 First Department of Internal Medicine, Tokyo Medical University, Tokyo, Japan; 2 Intractable Immune System Disease Research Center, Tokyo Medical University, 6-7-1, Nishi-shinjuku, Shinjuku-ku, Tokyo 160-0023, Japan; 3 Department of Virology, Medical Research Institute, Tokyo Medical and Dental University, Tokyo, Japan

**Keywords:** Epstein Barr virus, nasal NK/T-cell lymphoma, EBV DNA microarray, CAEBV infection

## Abstract

Nasal NK/T-cell lymphoma is an aggressive subtype of non-Hodgkin lymphoma (NHL) that is closely associated with Epstein–Barr virus (EBV). The clonal expansion of EBV-infected NK or T cells is also seen in patients with chronic active EBV (CAEBV) infection, suggesting that two diseases might share a partially similar mechanism by which EBV affects host cellular gene expression. To understand the pathogenesis of EBV-associated NK/T-cell lymphoproliferative disorders (LPD) and design new therapies, we employed a novel EBV DNA microarray to compare patterns of EBV expression in six cell lines established from EBV-associated NK/T-cell LPD. We found that expression of BZLF1, which encodes the immediate-early gene product Zta, was expressed in SNK/T cells and the expression levels were preferentially high in cell lines from CAEBV infection. We also analyzsd the gene expression patterns of host cellular genes using a human oligonucleotide DNA microarray. We identified a subset of pathogenically and clinically relevant host cellular genes, including TNFRSF10D, CDK2, HSPCA, IL12A as a common molecular biological properties of EBV-associated NK/T-cell LPD and a subset of genes, such as PDCD4 as a putative contributor for disease progression. This study describes a novel approach from the aspects of viral and host gene expression, which could identify novel therapeutic targets in EBV-associated NK/T-cell LPD.

Epstein–Barr virus (EBV) is a ubiquitous human herpesvirus that establishes a lifelong persistent infection of B cells in more than 90% of the human adult population (Cohen, 2000; Kieff and Rickinsonm, 2001) Primary infection by EBV is usually asymptomatic, however, it sometimes results in infectious mononucleosis (IM) which resolves spontaneously after the emergence of EBV-specific immunity (Cohen, 2000). EBV is a B-lymphotropic virus that is characterised by its ability to transform B cells *in vitro*, and is associated with a variety of B-cell malignancies including Burkitt lymphoma, non-Hodgkin lymphoma (i.e., pyothorax-associated lymphoma; PAL) and post-transplant lymphoproliferative disorders (LPD) (Nakatsuka *et al*, 2002; Thompson and Kurzrock, 2004). It is also demonstrated that EBV is implicated in the pathogenesis of non-B-cell malignancies, such as classical Hodgkin's lymphoma, NK- or T-cell lymphomas, nasopharyngeal carcinoma (NPC) and gastric carcinoma (Young and Rickinson, 2004).

Like other types of herpesvirus, EBV can infect cells in either a latent or a lytic manner. The EBV proteins associated with cellular transformation, such as LMP-1, are expressed in certain forms of latent infection, and most tumor cells in EBV-associated LPD express transformation-associated viral proteins (Huen *et al*, 1995; Zimber-Strobl *et al*, 1996; Kieff and Rickinsonm, 2001) Cellular transformation results from the latent rather than the lytic form of EBV infection, however, an increased level of lytically infected cells in patients may nevertheless increase the likelihood of an EBV-associated malignancy.

Although the molecular biology of EBV and B-cell transformation has been extensively studied for years, little is known about the host–virus interaction in EBV-related NK- and T-cell malignancies. Extranodal NK/T-cell, nasal type (hereafter, nasal NK/T-cell lymphoma), is a distinct clinicopathologic entity highly associated with EBV (Nava and Jaffe, 2005). Since necrosis is the common feature of nasal NK/T-cell lymphoma, the biological features of lymphoma cell are poorly understood because of the difficulty in obtaining sufficient viable specimens. Some nasal NK/T-cell lymphomas, develop from long-lasting EBV infection termed chronic active EBV (CAEBV) infection. CAEBV infection has high mortality and morbidity and its life threatening characteristics include virus-associated hemophagocytic syndrome and characterised by recurrent IM-like symptom (Kimura *et al*, 2001). The clonal expansion of EB infected NK- or T-cells is seen in patients with both nasal NK/T-cell lymphoma and CAEBV infection, suggesting that the two diseases might partly share a similar mechanism by which EBV affect host cellular genes. The question has thus arisen why a subset of patients with CAEBV infection develop nasal NK/T-cell lymphoma.

This study aimed to investigate the virus–host interaction in two types of EBV-associated NK/T-cell LPD, which may clarify the possible association between infection and cancer. We attempted transcriptional profiling of EBV genes using a novel viral DNA microarray, HHV-4 Viruchip®, in addition to host cellular gene expression profiling using human oligonucleotide DNA microarrays, seeking to evaluate the possible role of EBV–host interaction in nasal NK/T-cell lymphoma and CAEBV infection.

## MATERIALS AND METHODS

### Cells

We used six cell lines with EBV-associated T/NK-cell LPD: SNK-1^*^, SNK-6^*^ and SNT-8^*^ were isolated from nasal NK/T-cell lymphoma, and SNK-10, SNT-13 and SNT-16 were isolated from the peripheral blood of patients with CAEBV infection (Zhang *et al*, 2003). Asterisks were added to the designation of cell lines to easily distinguish those that were established from NK/T-cell lymphoma to those from patients with CAEBV infection. SNK-1^*^, SNK-6^*^, SNK-10 showed an NK cell phenotype, while SNT-8^*^, SNT-13, SNT-16 had a T-cell phenotype (Zhang *et al*, 2003). All the SNK/T cells had monoclonal EBV genomes identical to the original EBV-infected cells of the respective patients, and detailed characteristics of the cell lines were reported previously (Zhang *et al*, 2003). SNK/T cells were maintained in RPMI1640 with 10% heat-inactivated human serum and 700 U ml^−1^ of IL-2. The cell line, B95-8 was used as a positive control of lytic infection of EBV (Miller *et al*, 1972). A peripheral blood mononuclear cells obtained from a patient with acute phase of IM (IM-a cells), a Burkitt lymphoma cell line (Akata) and a EBV-transformed lymphoblastoid cell line (LCL-a) were also used (Shimizu *et al*, 1994). The original patient specimens from which IM-a and LCL-a established were acquired after obtaining written informed consent from the patients. A human lymphoid cell lines, Peer and WaY were used EBV negative controls (Ravid *et al*, 1980; Zhang *et al*, 2003).

### Quantification of EBV DNA copy number in SNK/T cells

DNA was extracted from both cells and the supernatants using a QIAamp Blood Kit (Qiagen, Basel, Switzerland). To determine whether encapsidated EBV rather than fragments of EBV DNA were present in culture supernatant of the SNK and SNT cell lines, 800 *μ*l of supernatant of cell lines was treated with or without DNase I (Invitrogen, Carlsbad, CA, USA) by which cell-free EBV DNA molecules are digested (Gallagher *et al*, 1999). Real-time quantitative polymerase chain reaction (PCR) was done using an ABI PRISM 7700 Sequence Detection System as reported previously (Aritaki *et al*, 2001).

### RNA isolation and cDNA labelling

Total RNA was extracted using a RNeasy Mini Kit (Qiagen, Germantown, MD, USA). For microarray analysis of host genes, mRNA was purified using an Oligotex™-dT30<Super>mRNA Purification Kit (Takara, Shiga, Japan). Total RNA (40 *μ*g) or 1 *μ*g of mRNA were reverse transcribed into cDNA and labelled by cyanine 5 (Cy5)-conjugated deoxyuracil triphosphate (dUTP) or cyanine 3 (Cy3)-conjugated dUTP (Amersham Biosciences, Piscataway, NJ, USA) using a LabelStar Array kit (Qiagen), according to the supplier's instruction. For the EBV DNA microarray, EBV-specific primer and control housekeeping gene specific primers (Celonex Inc., Edmonton, Alberta, Canada) were used instead of oligo-dT primer in the current study.

### EBV DNA microarray

The EBV DNA chip, HHV-4 Viruchip® (Celonex Inc.), was made up of triplicate spots of 70-mer oligonucleotide specific probes for each EBV transcription unit. The detailed information of the EBV DNA chip was deposited in an NCBI gene expression omnibus (GEO, accession number: GPL1917). Four human genes were included in order to standardise the amount of applied cDNA and to normalise the signals between samples and references. In all, 17 genes of HIV were also printed to exclude nonspecific hybridisation. For a dual colour analysis of EBV gene expression, the cDNA obtained from EBV-negative cells was labelled with Cy3 as a reference, and cDNA obtained from EBV-positive cells was labelled with Cy5. The EBV gene expression was then compared among various types of EBV infected cells. Hybridisation was performed following the same procedure as we recently reported in detail using the HHV-6B Viruchip® (Ohyashiki *et al*, 2005a). Briefly, HHV-4 Viruchip® was prehybridised in buffer containing 5 × SSC, 0.1% sodium dodecyl sulphate (SDS) and 1% BSA, then hybridised with labelled probes in ArrayHyb low-temperature hybridisation buffer (Sigma-Aldrich co., St Louis, MO, USA) containing 1/100 vol. of salmon testis DNA (Sigma-Aldrich Co.) at 42°C for 16 h. The HHV-4 Viruchip® was washed with microarray washing buffer (Sigma-Aldrich Co.) according to the manufacture's instruction.

### DNA microarray analysis of host cellular genes

To analyse host cellular gene expression patterns, we have designed two types of pathway focused low-density oligonucleotide microarrays (Novusgene Inc., Tokyo, Japan) which contains 528 selected genes related to cell growth, cell cycle, apoptosis, transcription, DNA repair and cell stress responses (GEO accession number: GPL1919 and GPL 1920). This DNA chip was made up of tetra-plicate spots of 60-mer highly specific oligonucleotide probes. For DNA microarray analysis of host genes, we used 1 *μ*g of mRNA obtained from cell lines. For dual colour analysis, normal human lymphocyte mRNA was purchased from Ambion (Austin, TX, USA), and utilised as a reference, and then cellular gene expression were compared among SNK/T cells. mRNA (1 *μ*g) was labelled with Cy-3 or Cy-5 and hybridisation was carried out automatically using GeneTAC Hybstation (Genomic Solutions, AnnArbor, MI, USA) according to the supplier's instruction. The conditions of hybridisation were 55°C for 2 h, 50°C for 2 h and 46°C for 2 h, then 42°C for 12 h.

### Data analysis and statistic validation

The hybridised signals were scanned by GenePix 4000B Microarray Scanner (Axon Instruments, Union City, CA, USA) as raw data. The scanned data were normalised, verified and analysed using the Genomic Profiler software® (Mitsui Knowledge Industry Inc., Tokyo, Japan) as reported previously (Ohyashiki *et al*, 2005a; Takaku *et al*, 2005). First, the value was adjusted by subtraction of background fluorescence of an equivalent area. Analysis was carried out by taking the median signal of the probe value for each transcript set, and the 75% rank for the total hybridisation was calculated. Microarray data obtained from three independent experiments were then verified in a single file. The normalised log data of fluorescence ratio (Cy5 to Cy3), which was quantified for each gene to reflect the relative abundance of gene in each experimental sample compared with reference sample, were deposited in GEO. To validate the amount of applied cDNA in EBV DNA microarray, we performed normalisation by five human housekeeping genes as reported previously, since we used uninfected cells as references (Ohyashiki *et al*, 2005a). This normalisation procedure is different from those used in human DNA microarray (global normalisation) (Takaku *et al*, 2005). For statistical analysis of host gene expression, we also utilised a GeneSifter® (VizXLabs, Seattle, WA, USA). Analysis of variance (ANOVA), and Student's *t*-test, were performed using GeneSifter®. Values of *P*<0.05 were considered to be indicate a statistically significant difference and theBenjamini-Hochberg algorithm was used for estimation of false discovery rates.

### Real-time Reverse transcriptase PCR (RT-PCR)

To confirm the results of microarray, we performed RT-PCR by ABI PRISM 7700 Sequence Detection System (Applied Biosystems, Foster City, CA, USA) as reported elsewhere (Ohyashiki *et al*, 2005b). For relative quantification of EBV gene expression, the level of gene expression was expressed as the percent of those in SNK-6^*^ cells.

For absolute quantification of BARF1, BHRF1 and BZLF1 gene expressions, we constructed plasmids that contain each amplified fragment using a pT7Blue T vector-2 kit (Novagen, Darmstadt, Germany) to generate a standard curve, then the amount of EBV gene expression in each sample was expressed as copy numbers per microgram of RNA with respect to the standard curve. Primers and probe sets for the EBV gene are described in Table 1.

For quantification of human gene expression, we utilised Taqman gene expression assays (Applied Biosystems), and the amount of gene expression in each sample was evaluated as a percent with respect to the standard curve generated from a serial dilution of quantitative PCR human reference total RNA (Stratagene, La Jolla, CA, USA).

### PCR direct sequencing of BARF1 gene

PCR-direct sequencing of BARF-1 was carried out using BigDye Terminator ver3.1 (Perkin-Elmer Cetus, Fremont, CA, USA). We designed four pairs of primers for nucleotide sequence analysis of BARF1. BARF1-1F: 5′-CCTCACA AACACAGAATCTG and BARF1-1R: 5′-GAGACCGAGGTCACCAAG; BARF1-2F:5′-CCGCTTTCTTGGGTGAGC and BARF1-2R: 5′-GCAAA TGGCGGTGTTATGAA; BARF1-3F: 5′-GTCACCGCTGCC AACATCTC and BARF1-3R: 5′TTGCGGGTGGATAGCCTC; BARF1-4F: 5′GTCTC AGTTC CCAGACTTCT and BARF1-4R: 5′AAATAGTTCCAGGTGACAGC.

### Expression of Zta in SNK/T cells

Western blot analysis for expression of Zta was performed according to the procedure previously reported (Zhang *et al*, 2003). Briefly, cells were washed twice in phosphate-buffered saline (PBS) and cell pellets were lysed in electrophoresis buffer and boiled for 10 min. The equivalent of 2.5 × 10^5^ cells was loaded onto each lane of an 8% Tris-glycine gel. The separated protein was blotted onto a filter and blocked in 5% skimmed milk, then incubated with Anti-Zta (Anti-Z Replication Activator) (Argene, Varilhes, FRANCE) in 5% skimmed milk in PBS for 90 min at room temperature. After washing, the blots were incubated for 30 min with horseradish peroxidase (HRP)-linked anti-mouse IgG. (Amersham Biosciences, Buckinghamshire, UK). Signals were visualised using a ECL Western blotting detection reagents and analysis system (Amersham Biosciences).

## RESULTS

### EBV virion was not detected in the supernatant of SNK/T cells

**Figure 1:**
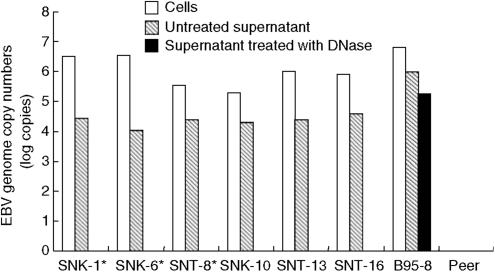
Quantification of EBV viral genome in SNK/T cells. The outlined bar indicates copy numbers of EBV in SNK/T cells. The hatched bar indicates the copy numbers of EBV in untreated supernatant. The black bar indicates the copy numbers of encapsidated EBV in DNase-treated supernatant that was only seen in B95-8 cells. In SNK/T cells, viral load is observed in both cells and untreated supernatant, whereas EBV DNA was undetectable after DNase treatment. Primers and probes are as follows: the forward primer; 5′-GGAACCTGGTCAT CCTTGC, the reverse primer; 5′-ACGTGCATGGACCGGTTAAT, and probe 5′-FAM- CG CAGGCACTCGTA CTGCTCGCT-TAMRA.

EBV load of the SNK/T cells were ranged from 3.4 × 10^5^ to 3.5 × 10^6^ copies *μ*g^−1^ of DNA (Figure 1). EBV DNA was also detected in the untreated supernatant of SNK/T cells; however, EBV DNA was undetectable in any supernatant after DNase digestion. This indicates that the EBV DNA present in the untreated supernatant of SNK/T cells consisted of naked DNA fragments rather than virions, which must be encapsidated. In contrast, the EBV copy numbers of B95-8 cells showing lytic infection was high in both cells and DNase-treated supernatant. Unlike in B95-8 cells, it is likely that active viral replication does not take place in SNK/T cells, although we could not completely rule out the possibility that only a small portion of SNK/T cells entered the lytic cycle which could not be detected by the current PCR assay. We did not detect any EBV in either cells or supernatant of Peer.

### EBV gene expression profiling using a EBV microarray, HHV-4 Viruchip®

**Figure 2:**
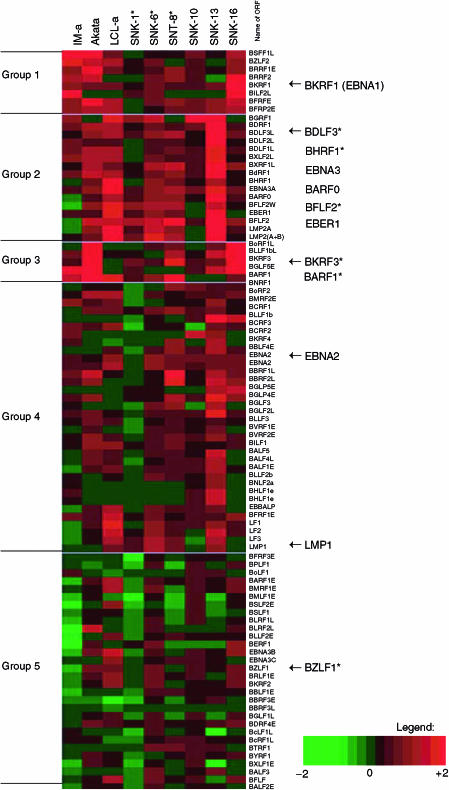
Transcriptional profiling of EBV in SNK/T cells and EBV associated diseases. The cluster analysis (K-mean) was done using Genomic Profiler® software. This colour map shows that genes whose expression is greater than EBV negative control is shown as red (see legend). The molecular signature of SNK/T cells is highly variable, but shares partly common features (see the text). Notable genes are indicated by arrowheads, and asterisks indicate lytic genes.

To compare the general gene expression pattern, we first investigated the transcriptional profiling of EBV using cells with various types of EBV infection. The tentative cluster analysis of EBV gene expression (K-mean, by Genomic Profiler®) showed marked heterogeneity among cell lines (Figure 2, See the GEO accession number, GSE2414 to retrieve all the data of EBV gene expression). The EBV gene expression pattern of cells from a patient in the acute phase of IM exhibited a distinct feature, which did not mimic any other samples used in the current study. As peripheral blood cells in IM patients may be composed of EBV lytically infected cells as well as latently infected cells (Bauer *et al*, 2005), we found that a subset of EBV lytic genes including BZLF1 in Group 5 were expressed in IM-a cells. In Akata cells, a limited number of latency-associated genes, such as BKRF1, which encodes EBNR1 (Group 1) was detected. Subsets of lytic gene such as BARF1 and BKRF3 (Group 3) were also expressed, which is consistent with the previous reports that a small portion of EBV positive Akata cells expressed lytic antigens (Sheng *et al*, 2003). In LCL-a, considerable numbers of both lytic and latent genes were expressed. Of note is that the expression profile of the above mentioned three cell lines showed its own distinct pattern of EBV gene expression.

In SNK/T cells, we found a wide variation of transcriptional profiles. The expression patterns of SNK-6^*^, SNT-8^*^ and SNT-13 somewhat mimic to those in LCL-a (genes in Group 3), while those cell line had monoclonal EBV genomes identical to the original EBV-infected cells of the respective patients. In contrast, the expression pattern of SNK-10 did not take after those of any other cell lines. The gene expression patterns in SNK/T cells, which were highly variable, shared some common characteristics. In general, the latent genes, such as BKRF1, LMP1, LMP2 (A+B), EBER1, EBER2 and BARF0 were detected in SNK/T cells. We could not find any difference of EBV gene expression between cells established from NK/T-cell lymphoma and those from CAEBV infection by EBV DNA microarray, however, it is notable that some lytic genes, such as BARF1, BKRF3, BDLF3, BFLF2 and BHRF1, were detected in most of the SNK/T cells.

### Quantitative relationship between EBV gene expression by microarray and a real-time RT-PCR

**Figure 3:**
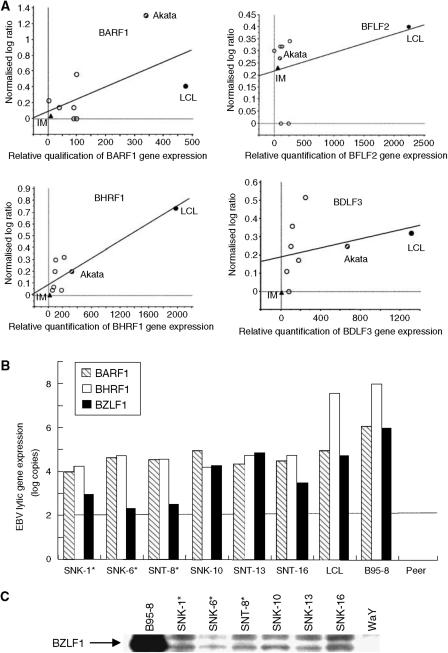
(**A**) Quantitative relationship between EBV gene expression by microarray and a real-time RT-PCR. Circles: SNK/T cells, Triangle: IM-a cells, and Solid dot: Akata. In general, the normalised log ratio of median was basically associated with the results obtained from real-time RT-PCR. When normalised log ratios were extremely high (i.e., LCL-a) or undetectable (i.e., IM-a), there was a significant correlation between the EBV gene expression levels by microarray analysis and those by a real-time RT-PCR. (**B**) Quantification of EBV gene expression by real-time RT-PCR. BARF1 is consistently expressed in all the SNK/T cells. The expression of BHRF1 is also seen in all the SNK/T cells, but the expression levels are approximately 2 log lower than those in LCL. (**C**) Western blot analysis of Zta in SNK/T cells. WaY was used as the negative control, and B95-8 was used as the positive control. All the SNK/T cells were weakly positive for Zta.

In the current study, we found some discrepancy between data obtained from the EBV microarray and our previous observations using RT-PCR and Western blotting. For instance, the expression of LMP-1 gene in SNK-10 was negative by EBV DNA microarray despite the fact that our previous study showed LMP1 expression by Western blotting (Zhang *et al*, 2003). Since we used EBV uninfected cell as the reference, value 0 (normalised log 2 ratio) means the background subtracted fluorescence intensities after careful normalisation of the amount of applied cDNA may be equal in the sample and the references. It was possibly due to nonspecific hybridisation. We, therefore, arbitrarily determined the cutoff level as 1.5, however, unlike with human DNA microarray, it was rather difficult to determine the appropriate cut-off value in EBV DNA microarray. To validate the results obtained from the HHV-4 Viruchip®, we therefore compared the normalised log 2 ratio of the median calculated by microarray analysis and the relative expression level of EBV by a real-time RT-PCR. The normalised log ratio of the median was associated with the results obtained from real-time RT-PCR, although we found some discrepancy between data obtained from the two methods especially when the normalised log ratio was relatively low (Figure 3A). In contrast, we found significant correlation between the EBV gene expression levels by microarray analysis and those by real-time RT-PCR, when normalised log ratios were extremely high (i.e., LCL-a) or undetectable (i.e., IM-a).

### BARF1, BHRF1 and BZLF1 expression in SNK/T cells infection

Our previous study mainly focused on the latent gene expression in SNK/T cells (Zhang *et al*, 2003), however, the transcriptional profiling of EBV led us to consider several lytic genes which may play some role in the development of EBV infected SNK/T cells. The quantitative real-time RT-PCR revealed that BHRF1 was expressed in SNK/T cells, but the expression was the lower at a 2 log scale compared to B95-8 and LCL-a. In contrast, the level of BARF1 gene expression in SNK/T cells was equivalent to that in LCL. We, therefore, sought to determine the sequence variants within the BARF1 sequences by PCR-direct sequencing. We found several sequence variations with respects to the sequence of B95-8 (NC_001345) within the BARF1 (i.e., G to A at the position of 165554 and T to C at 165589 in SNK-1 and SNK-8; T to C at 165545 in SNT-16). Although the expression level of BZLF1, which encodes the immediate-early gene products Zta detected by DNA microarray was relatively low in SNK/T cells, real-time RT-PCR showed that the expression of BZLF1 was detected in all the SNK/T cells (Figure 3B). It is noteworthy that the copy numbers of BZLF1 in SNK/T cells derived from CAEBV infection was significantly higher than those from NK/T-cell lymphomas (*P*=0.049). Western blot analysis for the expression of Zta showed that all the SNK/T cells were positive for Zta, confirming the results obtained from real-time RT-PCR (Figure 3C).

### The common molecular signature of host cellular genes in SNK/T cells

**Figure 4:**
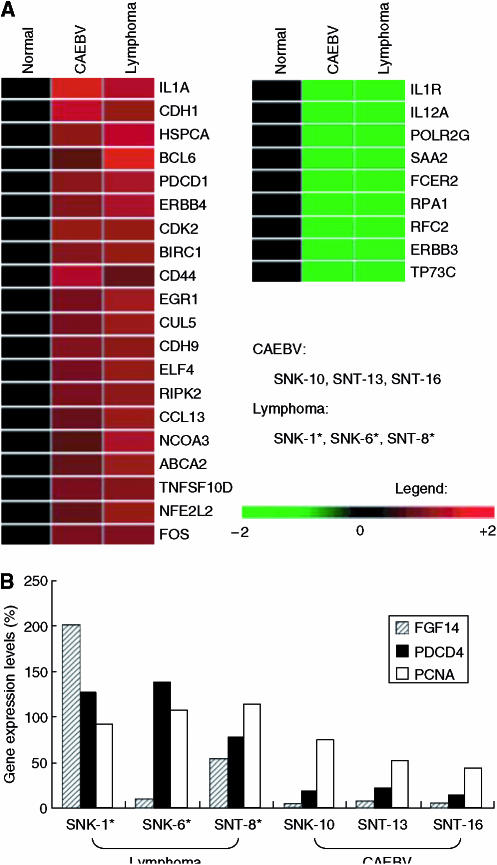
(**A**) The common molecular signature of SNK/T cells. ANOVA analysis was performed by GeneSifter® using microarray analysis data deposited in GEO (GSE2414). Genes whose expression levels are 4-fold greater (left) or lower (right) in SNK/T cells compared to those in normal lymphocytes were navigated by GeneSifter®. A list of gene names is also shown in Table 2. (**B**) Genes preferentially expressed in lymphoma cell lines. Quantitative real-time RT PCR of FGF14, PDCD4, and PCNA in SNK/T cells. The expression levels are higher in cell lines established from NK/T lymphoma consistent with the results obtained from microarray analysis.

In SNK/T cell lines, selection of clones with growth advantage *in vitro* might occur during the immortalisation process, despite being established in a clinically different disease. We first investigated the common molecular signature of SNK/T cells (to retrieve all the data of normalised log ratio, see GEO accession number: GSE2414). Differential expression was analysed using a GeneSifter® (Figure 4A). Genes with elevated expression levels in SNK/T cells (expression level in the sample was four-fold greater than in control lymphocytes) were listed in Table 2A. We found that a subset of genes, which may play regulatory roles in apoptosis, including IL1A, BCL6, TNFRSF10D, RIPK2 and PDCD1, was upregulated in SNK/T cells. It is notable that upregulation of CDK2, which is related to cell cycle regulation, HSPCA, encoding heat shock protein 90 and ELF4, which may be linked to NK/T cell proliferation, were common biological characteristics of SNK/T cells. Genes related to cell adhesion, such as CDH1, CD44, CUL5 and CDH9 were also upregulated in SNK/T cells. In contrast, a subset of genes, such as IL12A, which is known to be an interferon-*γ* inducer, was remarkably downregulated (expression level in the sample was four-fold lower than in control lymphocytes) in SNK/T cells (Table 2B).

### The difference of host gene expression between cell lines established from NK/T lymphoma and those from CAEBV infection

To ascertain whether the host cellular gene expression of SNK/T cells established from nasal NK/T-cell lymphoma patients is different from that of cells established from CAEBV infection patients, we next compared the gene expression pattern between two groups, one with cells from NK/T-cell lymphoma and the other from CAEBV infection. Eventually, we found several genes with expression levels significantly higher in cell lines established from NK/T-cell lymphoma than those from CAEBV infection (Table 3): FGF14, PDCD4, PCNA, MAP2K4, ITGAX and AKAP2 were preferentially expressed in SNK/T cells established from nasal NK/T lymphoma. The expression levels of genes, FGF14, PCNA and PDCD4 were compatible with the results obtained from real-time RT-PCR (Figure 4B).

## DISCUSSION

This may be the first report dealing with the molecular signature of EBV in nasal NK/T-cell lymphoma and CAEBV infection. Moreover, our DNA microarray approach from aspects of viral and host gene expression might provide insight into the complex genetic process underlying the interaction between EBV and NK/T-cells. Evidence suggests that the oncogenic potential of EBV lies largely in a set of cell cycle promoting EBV genes that are expressed during latency, therefore, latency associated genes, such as LMP1, are likely to play a major role in the viral life cycle by causing amplification and distribution of infected cells (Huen *et al*, 1995; Zimber-Strobl *et al*, 1996). In contrast, recent studies demonstrated that herpesviruses, such as the human cytomegalovirus, which belongs to the *β*-herpesviruses, can actively block cell cycle progression during lytic infection, indicating that viral replication suppresses cell growth and vice versa (Wei *et al*, 1994; Jault *et al*, 1995; Lu and Shenk, 1996). The same argument can be applied to EBV infection.

A group of abundantly expressed RNAs that are encoded by the *Bam*HI region of the EBV genome were originally identified in NPC (Young and Rickinson, 2004). Although there were more than 20 *Bam*HI fragments in EBV, we specially marked down BARF1, which is generated from the *Bam*HI fragment region, originally identified as an early antigen expressed on induction of the EBV lytic cycle. BARF1 shares limited homology to intracellular adhesion molecule 1 (ICAM-1) and human colony stimulating factor 1 receptor (hCSF-1 receptor) and display oncogenic activity in both EBV negative Akata cells and human Louckes B-lymphocytes (Sheng *et al*, 2003)). Recent studies demonstrated that BARF1 is a secreted protein that is expressed as a latent protein in EBV-associated NPC and gastric carcinoma (Decaussin *et al*, 2000; zur Hausen *et al*, 2000). In the present study, we found that BARF1 was expressed in all the SNK/T cells. While the pathological significance of the sequence variations within BARF1 remains uncertain, we postulate that BARF1 expression might be linked to the pathogenesis in NK/T-LPD, in keeping with its role in NPC (Decaussin *et al*, 2000).

In the current study, we also found expression of several EBV lytic genes (i.e., BHRF1 which shows partial sequence homology to the human bcl-2 proto-oncogene, BKRF3, BDLF3 and BFLF2) in all of the SNK/T cells. Despite the fact that SNK/T cells express lytic genes, we could not find any detectable amount of virion DNA in the supernatant of SNK/T cells, suggesting that the vast majority of SNK/T cells have abortive EBV infection. Of note is that the BZLF1 was detected in all the SNK/T cells by quantitative RT-PCR and we confirmed expression of Zta by a Western blotting analysis. A recent study by Kimura *et al* (2005) demonstrated that patients with T-cell type CAEBV infection had higher titers of immunoglobulin G against early EBV antigen, suggesting lytic cycle infection. Taken together with our results, EBV lytic gene may play an important role in the pathogenesis of NK/T-cell LPD.

Probably because of the technical pitfalls of viral microarray analysis (Ohyashiki *et al*, 2005a), some discrepancy was seen in the genes detected by microarray analysis compared with the results obtained from RT-PCR studies. Since the sensitivity of the DNA microarray seemed to be different from that of conventional assays, we conclude that EBV microarray technology is suitable for determining global gene expression patterns in EBV-related diseases but inappropriate for genes especially when EBV gene expression was low. The discrepancy should be resolved by increasing the resolution of microarrays, by extensive and technical repeats of hybridisation, by using proper reference, by performing an appropriate normalisation procedure and by carefully characterising the nature of viral transcriptome. The primary goal of this study, the transcriptional profiling of EBV, might provide new insight into the EBV gene expression of T/NK-cell LPD, which traditionally has been categorised as latency type II infection. While the use of EBV microarrays is still in its infancy, this emerging technology will improve our understanding of host-pathogen interaction.

The secondary goal of this study was to investigate the common molecular properties of SNK/T cells. Mainly due to the heterogeneity of biological properties among SNK/T cells, we found great variations among cell lines regardless of the cell lineage. We intended to focus the genes in SNK/T cells whose expression level in the sample was four times greater than in control lymphocytes. Theoretically, the comparison of cells of the same lineage at a similar differentiation stage would be ideal for human gene expression analysis, however, in practical terms this was difficult to perform, since samples of both T and NK lineage were included in this study. Even though all the SNT series are of T-cell lineage, SNT-8, and -13 have *γδ*T-cell phenotype and SNT-16 has *γδ* T-cell phenotype. We, therefore, used normal lymphocytes as the same reference RNA for dual colour microarray analysis, and then compared the host gene expression levels in both T- and NK-cell lines. It is notable that the expression level of TNFRSF10D, which inhibits toll-like receptor (TRAIL) mediated apoptosis, was significantly higher in SNK/T cells. A recent study demonstrated that a lytic EBV gene, BHRF-1 drastically inhibits TRAIL-mediated apoptosis in BJAB cells (Kawanishi *et al*, 2002). Matrix-based comparative genomic hybridisation (matrix-CGH) analysis demonstrated that TNFRSF10D is one of the pathologically relevant genes in mantle cell lymphoma (Kohlhammer *et al*, 2004). In addition, upregulation of RIPK2, which accelerates the NF*κ*B and MAPK pathways, appears to be linked to antiapoptotic effect in SNK/T cells. Therefore, we reasoned that antiapoptotic pathways might be involved to the virus–host interaction in SNK/T cells whether or not cells were derived from NK/T-cell lymphoma.

Another important issue that we should address is that the expression of CDK2, which is considered to be a key cell-cycle regulatory factor, was upregulated in SNK/T cells. Interestingly, the expression of BZLF1, which encodes Zta was high in a subset of SNK/T cells. Zta may carry out its function through a complex series of interactions with multiple cell cycle control proteins, such as p53 and pRb (Rodriguez *et al*, 2001; Lin *et al*, 2004) Obviously, it must be clarified whether the Zta-mediated cell cycle control mechanism in SNK/T cells is similar to that reported in other types of EBV-related B-cell malignancies. We also found overexpression of HSPCA, which encodes heat shock protein 90, which has recently been reported as a pathologically relevant gene candidate in mantle-cell lymphoma by proteomic analysis (Ghobrial *et al*, 2005). Marked downregulation of IL12A in SNK/T cells allowed us to consider the possibility to adoptive immunotherapy with EBV-specific cytotoxic-T-lymphocytes (Robertson *et al*, 2005).

The clonal expansion of EBV-infected T or NK cells is a common feature seen in both NK/T-cell lymphoma and CAEBV infection. Although SNK/T cells share common biological properties, statistical analysis using a GeneSifter® revealed that a subset of genes including PDCD4 may contribute to the development of lymphoma. We could not completely exclude the possibility that this was possibly due to *in vitro* selection of EB virus infected NK or T cells, therefore, further studies using samples obtained from patients will be required to rigorously address this issue.

Although the number of cell lines at hand was limited in the current study, our results convincingly suggest the possibility that certain EBV genes, such as BZLF1 could be a therapeutic target for EBV-related NK/T-cell LPD. Moreover, our data provide a basis for further studies on the identification of pathogenically and clinically relevant host cellular genes, such as TNFRSF10D, CDK2, Hsp90, IL12A and PDCD4. Since the biology of EBV infection *in vivo* still remains unclear, to investigate the molecular signature of both virus and host might introduce a novel target for antineoplastic therapies including vaccines.

## Figures and Tables

**Table 1:** Sequences of primers and probes for real-time RT-PCR

**Target**	**Oligo**	**Sequence: Genebank accession #NC_001345**	**Coordinates**
BARF1	Forward primer	GGTGAAGCCTCTAACGCTGTCT	165866–165887
	Reverse primer	TCACGGTGCATGTCACAGTAAG	165927–165948
	Probe	TCCACTCCGAAAGGTCTCAGTTCCCAG	165889–165915
BHRF1	Forward primer	CAGGACATTGTGTTGTAACCAGTCT	54711–54735
	Reverse primer	GCCTTCGCTGGCTTCTAACAT	54772–54792
	Probe	CTCCTTACTATGTTGTGGACCTGTCAGTTCGTG	54737–54769
BDLF3	Forward primer	TAGTCGTGGTCAAGGTCGTAGACT	130874–130897
	Reverse primer	ACAACGGCTGTGACTACACCAT	130928–130949
	Probe	TGCTAGGACCCGATGCCGACG	130904–130924
BFLF2	Forward primer	GCACATGAACCTGGGACCTATT	56467–56488
	Reverse primer	CTTCGGCCACAGCCTGACT	56519–56534
	Probe	ATGCAGATCTCGCAGTGAGACCCCA	56490–56514
BZLF1	Forward primer	GGAGGAATGCGATTCTGAACTAG	102449–102466
	Reverse primer	GCTTAAACTTGGCCCGGC	102506–102528
	Probe	TTTTCTGGAAGCCACCCGATTCTTGTA	102468–102494

**Table 2:** Common molecular signature in SNK/T cells derived from both nasal NK/T lymphoma and CAEBV

**GeneBank accession #**	**Gene name**		**Molecular function**
(A) Upregulated genes whose expression level was greater than four-fold compared to those in normal lymphocytes			
NM_000575	IL1A	Interleukin 1, alpha	Negative regulation of apoptosis, innate immune response
NM_004360	CDH1	Cadherin 1, type 1, E-cadherin (epithelial)	Cell adhesion
NM_005348	HSPCA	Heat shock 90kDa protein 1, alpha	Cell stress
NM_001706	BCL6	B-cell CLL/lymphoma 6 (zinc finger protein 51)	Regulation of cell proliferation
NM_005018	PDCD1	Programmed cell death 1	Response to pest/pathogen/parasite
NM_005235	ERBB4	v-erb-a erythroblastic leukemia viral oncogene homolog 4	Signal transduction
NM_001798	CDK2	Cyclin-dependent kinase 2	Cell cycle, regulated by CDK inhibitors, such as p21Cip 1(CDKN1A), p27Cip 1(CDKN1B)
NM_004536	BIRC1	Baculoviral IAP repeat-containing 1	Negative regulation of apoptosis
XM_030326	CD44	CD44 antigen	Extracellular matrix
NM_001964	EGR1	Early growth response 1	Transcription
NM_003478	CUL5	Cullin5	Extracellular matrix
NM_016279	CDH9	Cadherin 9, type 2 (T1-cadherin)	Cell adhesion
NM_001421	ELF4	E74-like factor 4 (ets domain transcription factor)	Transcription, DNA-dependent, NK T-cell proliferation
NM_003821	RIPK2	Receptor-interacting serine-threonine kinase 2	Apoptosis, activation of MAPK pathway and NF kappa B pathway
NM_005408	CCL13	Chemokine (C-C motif) ligand 13	Innate immune response
NM_006534	NCOA3	Nuclear receptor coactivator 3	Transcription, DNA-dependent
NM_001606	ABCA2	ATP-binding cassette, sub-family A (ABC1), member 2	Metabolism
NM_003840	TNFRSF10D	Tumor necrosis factor receptor superfamily, member 10d	Negative regulation of apoptosis
NM_006164	NFE2L2	Nuclear factor (erythroid-derived 2)-like 2	Transcription, DNA-dependent
NM_005252	FOS	v-fos FBJ murine osteosarcoma viral oncogene homolog	Innate immune response, cell growth
			
(B) Downregulated genes whose expression level was lower than 4.0 fold compared to those in normal lymphocytes.			
NM_000877	IL1R1	Interleukin 1 receptor, type I	Response to pest/pathogen/parasite
NM_000882	IL12A	Interleukin 12A (natural killer cell stimulatory factor 1) (IL12A),	T-cell independent induction of interferon (IFN)-gamma
NM_002697	POLR2G	POU domain, class 2, transcription factor 1	DNA repair
NM_030754	SAA2	Serum amyloid A2	Extracellular matrix
NM_002002	FCER2	Fc fragment of IgE, low affinity II, receptor for (CD23A)	Immunoglobulin binding
NM_002945	RPA1	Replication protein A1, 70 kDa	Response to DNA damage stimulus
NM_002914	RFC2	Replication factor C (activator 1) 2, 40kDa	DNA replication
NM_001982	ERBB3	v-erb-b2 erythroblastic leukemia viral oncogene homolog 3	Metabolism
NM_005427	TP73	Tumor protein p73	Induction of apoptosis by intracellular signals

ANOVA analysis was performed by GeneSifter using microarray analysis data deposited in GEO (GSE2414). Statistically significant (*P*<0.05) genes were listed above.

**Table 3:** Genes preferentially expressed in cell lines derived from nasal NK/T lymphoma (SNK-1^*^, SNK-6^*^, SNT-8^*^) compared to those with CAEBV (SNK-10, SNT-13, SNT-16)

**GeneBank accession #**	**Gene name**	**Molecular function**	***P*-value**
NM_004115	FGF14	Growth factor activity	6.75E-06
NM_014456	PDCD4	Cell death	0.001200532
NM_002592	PCNA	Cell growth	0.001395258
NM_007203	AKAP2	Signal transduction	0.001985058
NM_000887	ITGAX	Cell adhesion, integrin-mediated signaling pathway	0.02959104
NM_003010	MAP2K4	Protein modification	0.030605858
NM_003250	THRA	Regulation of transcription	0.030747275
NM_001089	ABCA3	Cellular process	0.042526959
NM_002446	MAP3K10	Positive regulation of apoptosis, activation of JNK activity	0.042943068

Pairwise analysis (*t*-test) was performed by GeneSifter using microarray analysis data deposited in GEO (GSE2414): Group 1: Cell lines derived from CAEBV, Group 2: Cell lines derived from NK/T-cell lymphoma. Correction was done by Benjamin Hochberg method.
